# Sun-protective behaviours in US adults with a history of skin cancer: a cross-sectional National Health Interview Survey study

**DOI:** 10.1093/skinhd/vzag024

**Published:** 2026-05-15

**Authors:** Hannah Verma, Benjamin Hu, Nithya Krishnamurthy, Annie Chang, Raphaella Lambert, Grace Rabinowitz, Nicholas Gulati, Jonas Adalsteinsson

**Affiliations:** Kimberly and Eric J. Waldman Department of Dermatology, Icahn School of Medicine at Mount Sinai, New York, NY, USA; Kimberly and Eric J. Waldman Department of Dermatology, Icahn School of Medicine at Mount Sinai, New York, NY, USA; Kimberly and Eric J. Waldman Department of Dermatology, Icahn School of Medicine at Mount Sinai, New York, NY, USA; Kimberly and Eric J. Waldman Department of Dermatology, Icahn School of Medicine at Mount Sinai, New York, NY, USA; Kimberly and Eric J. Waldman Department of Dermatology, Icahn School of Medicine at Mount Sinai, New York, NY, USA; Kimberly and Eric J. Waldman Department of Dermatology, Icahn School of Medicine at Mount Sinai, New York, NY, USA; Kimberly and Eric J. Waldman Department of Dermatology, Icahn School of Medicine at Mount Sinai, New York, NY, USA; Department of Medicine, University of Iceland, Reykjavík, Iceland; Department of Dermatology, Landspitali University Hospital of Iceland, Reykjavík, Iceland

## Abstract

**Background:**

Individuals with a history of skin cancer are at elevated risk for subsequent malignancies, underscoring the importance of sustained photoprotective behaviours. However, little is known about how these behaviours evolve over time following diagnosis.

**Objectives:**

To assess the prevalence of sun-protective behaviours among adults with and without a history of skin cancer and to evaluate whether adherence varies based on time since diagnosis.

**Methods:**

We conducted a cross-sectional analysis using pooled data from the 2005–15 National Health Interview Survey. Weighted estimates were used to compare photoprotective behaviours, including sun avoidance, use of protective clothing, sunscreen application, use of tanning devices and sunburn history, between respondents with and without a history of skin cancer, stratified by years since diagnosis. Multivariate logistic regression was used to assess adjusted odds ratios (aORs).

**Results:**

Individuals with a history of skin cancer reported significantly higher use of sunscreen and protective clothing, and lower use of tanning devices than controls. Behaviours varied over time, and those with 1–4 years or ≥10 years since diagnosis had a significantly higher odds of using protective clothing compared with controls (adjusted OR 3.17, 95% confidence interval 1.35–7.46 for 1–4 years; adjusted OR 2.42, 95% confidence interval 1.22–4.79 for ≥10 years).

**Conclusions:**

Photoprotective behaviours varied by time since diagnosis, with peak adherence seen 1–4 years postdiagnosis. Photoprotection improves after skin cancer diagnosis but may change over time, supporting the need for ongoing patient education.

## Teaser

This study analysed data from the National Health Interview Survey to assess sun protective behaviours among adults with and without a history of skin cancer, stratified by time since diagnosis. Individuals with prior skin cancer reported greater use of sunscreen and protective clothing; however, behaviours were not consistent over time. These findings highlight the need for ongoing sun-safety counselling.

What is already known about this topic?

- Individuals with a history of skin cancer engage in more sun-protective behaviours than those without a history of skin cancer, yet overall adherence remains suboptimal.

What does this study add?

- This study shows that photoprotective behaviours are highest in the first few years following diagnosis but may decline over time.
- The results highlight the need for ongoing education and reinforcement of sun safety.

The incidences of basal cell carcinoma (BCC), squamous cell carcinoma (SCC) and melanoma have continued to rise over the past several decades.^1^ Individuals diagnosed with one form of skin cancer are at increased risk of developing subsequent skin cancers, regardless of the primary skin cancer type.^2,3^ This elevated risk is primarily attributed to cumulative ultraviolet (UV) radiation exposure and prior sunburns, with UV-induced skin damage being largely irreversible.^4,5^ Among patients with melanoma, the risk of developing a second melanoma is nine times higher than in the population without melanoma, and the risk of subsequent BCC or SCC is also significantly elevated.^2,6^

While UV exposure is a well-established, modifiable risk factor, relatively little is known about how long-term sun-protection behaviours evolve over time following a skin cancer diagnosis. Prior studies suggest that individuals with a history of skin cancer may adopt more protective behaviours.^6^ Survivors of melanoma report lower weekday sun exposure, fewer sunburns and reduced indoor tanning compared with control individuals, although weekend sun exposure remains similar.^5^ Increased sun protection in survivors of melanoma correlates with greater knowledge and worry about melanoma.^7^

Adults with prior nonmelanoma skin cancer also demonstrate higher rates of protective behaviours such as wearing long sleeves and wide-brimmed hats, seeking shade and using sunscreen, although they report no significant difference in sunburn incidence compared with the general population.^4^ Despite these efforts, approximately 30% of people with a history of skin cancer lack consistent sun protection.^4^

Importantly, overall health behaviours in survivors of cancer often change over time. In one study of Australian women, multiple health behaviour scores were highest in the years immediately following a cancer diagnosis, including habits such as increased physical activity and better dietary choices.^8^ However, these scores tended to decline over time, particularly beyond 3 years.^8^ This raises the question of whether sun-protection habits similarly diminish with time since diagnosis, representing a critical area of study. Survivorship after keratinocyte carcinomas continues to improve, with studies showing no significant reduction in 5-year survival after diagnosis with BCC or SCC *in situ*.^9^

In this study, we used data from the National Health Interview Study (NHIS) to examine sun-protection behaviours among individuals with a history of skin cancer, stratified by time since diagnosis, and to compare these findings against the population without a history of skin cancer. These findings may help inform long-term counselling strategies and identify the subgroups in most need of behavioural reinforcement.

## Patients and methods

The NHIS is conducted by the US Department of Health and Human Services, and is an annual cross-sectional survey of health conditions, behaviours and access that is nationally representative of the US civilian population. Participants are not followed longitudinally or repeated across survey years, and all data are self-reported. Data were pooled from the 2005, 2008, 2010 and 2015 NHIS records. These years were selected due to the comprehensive collection of data regarding multiple types of sun-protective behaviours. In 2019, the NHIS underwent a major redesign, and the 2020 NHIS did not collect complete data on protective clothing use and thus was not comparable with prior years. No data were collected on any sun-related behaviours after 2020. This study included all sampled adults from the pooled survey years who provided responses regarding sun-protective behaviours, tanning device use and sunburns. Respondents with missing data for any of the assessed variables were excluded for complete-case analysis.

All sample adult respondents from each survey administration were asked, ‘Have you ever been told by a doctor or other health professional that you had cancer or a malignancy of any kind?’ As a follow-up, sample adults were asked ‘What kind was it?’ Respondents were categorized as having a history of skin cancer if they reported any kind of skin cancer (melanoma, nonmelanoma skin cancer or unknown type of skin cancer) and they were diagnosed ≥1 year previously. If they reported some kind of skin cancer, they were included in the skin cancer group, while those who reported no history of skin cancer were included in the reference group. Respondents were also asked about their age at the time of each diagnosis.

Time since diagnosis was calculated by subtracting the age at the time of first diagnosis of skin cancer from the respondent’s age reported at the time of participating in the survey. Respondents with a history of skin cancer were stratified by time since diagnosis at the time of the survey (1–4 years, 5–9 years, ≥10 years), with no overlap between the groups.

Outcome variables in this study included self-reported frequency of sun-protective behaviours on a sunny day when outside for >1 h, including use of sun-protective clothing (hat, long sleeves, long pants), sunscreen use, and sun avoidance or shade seeking. Other outcome variables included the number of times a tanning device was used over the last 12 months and the number of sunburns over the last 12 months. All outcome variables were transformed into binary variables (1 and 0) to reflect regular engagement vs. minimal or no engagement for analysis, an approach previously used in other NHIS studies. For all outcomes pertaining to behaviours on a sunny day, the responses, ‘always’, ‘most of the time’ and ‘sometimes’ were grouped together (quantified as a value of 1), while the responses ‘rarely’ and ‘never’ were grouped together (quantified as a value of 0). For counts of using tanning devices over the last 12 months and sunburn over the last 12 months, all responses with a value ≥1 were categorized as ‘yes’ (quantified as a value of 1). Full distributions of responses across categories are provided in Table S1 (see Supporting Information).

Prevalence estimates and odds ratios (ORs) incorporated NHIS sampling weights for sample adults to account for the complex multistage survey design, which includes stratification and clustering; application of these weights adjusts for differing probabilities of selection and nonresponse to produce estimates that are representative of the US civilian adult population.^10^ Rao–Scott χ^2^-tests were used to compare prevalences across groups using ­survey-weighted estimates. Multivariate logistic regression was used to analyse associations between skin cancer diagnosis status and prevalence of outcomes. Multivariate logistic regression models were additionally adjusted for the following covariates: age, sex, race or ethnicity, region, health insurance, alcohol use, smoking status, education and income. Covariates were selected based on prior literature.

There were significant demographic differences between the reference cohort and the cohort with a history of skin cancer: 3.2% of the group with skin cancer was not White, compared with 16.0% of the cohort with skin cancer. Therefore, the analyses were repeated after restricting the sample to non-Hispanic White respondents as part of a secondary sensitivity analysis.

Data were analysed using the ‘survey’ and ‘srvyr’ packages in R version 4.4.1 (R Foundation, Vienna, Austria). *P*-values <0.05 were considered statistically significant in two-sided tests.

## Results

The weighted sample included a total of 61 383 730 adults, of whom 306 468 (0.5%) had reported a previous skin cancer diagnosis. There was no significant difference in age between the reference group and groups with a history of skin cancer, although there were significant differences in race, income level and educational attainment between the groups (Table 1).

**Table 1:** Demographics of respondents, National Health Interview Survey 2005–15 (weighted)

	No history of skin cancer diagnosis(*n* = 61 383 730)	History of skin cancer: time since diagnosis			*P*-value
		1–4 years(*n* = 115 743)	5–9 years(*n* = 43 854)	10–100 years(*n* = 146 871)	
Age (years), mean (SD)	41.8 (0.15)	48.6 (3.36)	53.1 (3.64)	55.2 (2.50)	0.05
Sex					<0.01
Male	28 027 552 (45.7)	46 717 (40.4)	28 514 (65.0)	68 403 (46.6)	
Female	33 356 178 (54.3)	69 026 (59.6)	15 340 (35.0)	78 468 (53.4)	
Race and ethnicity^a^					<0.01
White	51 536 361 (84.0)	114 022 (98.5)	43 854 (100.0)	138 774 (94.5)	
Black	6 139 930 (10.0)	1721 (1.5)	0	2926 (2.0)	
Asian	2 104 663 (3.4)	0	0	0	
Other	1 602 776 (2.6)	0	0	5171 (3.5)	
Education					<0.01
High school or less	16 593 701 (27.0)	26 232 (22.7)	4455 (10.2)	38 748 (26.4)	
Some college	22 641 494 (36.9)	42 767 (36.9)	27 534 (62.8)	52 461 (35.7)	
College graduate or above	22 148 535 (36.1)	46 744 (40.4)	11 865 (27.1)	55 662 (37.9)	
Income (USD)					<0.01
<$45 000	40 076 864 (65.2)	75 682 (65.4)	18 144 (41.4)	95 268 (64.9)	
>$45 000	21 306 866 (34.7)	40 061 (34.6)	25 710 (58.6)	51 603 (35.1)	
Region					<0.01
Northeast	9 384 302 (15.3)	33 000 (28.5)	9757 (22.2)	12 386 (8.4)	
Midwest	16 450 068 (26.8)	37 016 (32.0)	5723 (13.1)	40 976 (27.9)	
South	21 666 089 (35.3)	15 311 (13.2)	14 337 (32.7)	49 025 (33.4)	
West	13 883 271 (22.6)	30 416 (26.3)	14 037 (32.0)	44 484 (30.3)	
Health insurance					<0.01
Yes	51 107 057 (83.3)	105 206 (90.9)	39 782 (90.7)	129 675 (88.3)	
No	10 195 805 (16.7)	10 537 (9.1)	4072 (9.3)	17 196 (11.7)	
Unknown	80 868 (0.1)	0	0	0	
Smoking status					<0.01
Currently smokes	12 741 555 (20.7)	23 539 (20.3)	14 072 (32.1)	29 763 (20.3)	
Formerly smoked	12 407 011 (20.2)	18 579 (16.1)	13 786 (31.4)	49 612 (33.8)	
Never smoked	36 199 773 (59.0)	73 625 (63.8)	15 996 (36.5)	67 496 (46.0)	
Unknown	35 391 (0.1)	0 (0.0)	0 (0.0)	0 (0.0)	
Alcohol use					<0.01
Often	2 835 806 (4.6)	24 112 (20.8)	5472 (12.5)	10 618 (7.2)	
Semi-often	5 964 921 (9.7)	0	12 135 (27.7)	8577 (5.8)	
Not often	45 070 141 (73.4)	83 113 (71.8)	26 247 (59.9)	114 744 (78.1)	
Other	7 512 862 (12.2)	8518 (7.4)	0	12 932 (8.8)	

The data are presented as *n* (%) unless stated otherwise. Respondents were categorized into mutually exclusive groups based on skin cancer history and time since first diagnosis. Values are weighted to represent the US adult population. *P*-values represent overall comparisons of demographic characteristics across groups using survey-weighted χ^2^ tests for categorical variables and survey-weighted linear regression for age. ^a^Race categories were derived from National Health Interview Survey (NHIS) response options. ‘Other’ includes participants categorized by NHIS as American Indian/Alaska Native, multiracial, other race, race group not releasable or unknown/refused/not ascertained/don’t know.

When comparing all adults with a history of skin cancer vs. those without, the prevalence of protective clothing use was significantly higher in the group with a history of skin cancer [39%, 95% confidence interval (CI) 27–51 vs. 18%, 95% CI 17–19; *P* < 0.01], as was the prevalence of sunscreen use [71% (95% CI 60–82) vs. 59% (95% CI 58–60); *P* = 0.02]. There was significantly lower tanning bed use in those with a history of skin cancer [5% (95% CI 0–10) vs. 11% (95% CI 10–11); *P* = 0.02] (Table 2).

**Table 2:** Sun-protective behaviours among adults with and without a history of skin cancer, pooled across all years since diagnosis, National Health Interview Survey 2005–15 (weighted)

Behaviour	Individuals with a history of skin cancer^a^	Individuals without a history of skin cancer	*P*-value
Sun avoidance	78 (68–88)	70 (69–71)	0.1
Protective clothing	39 (27–51)	18 (17–19)	**<0.01**
Sunscreen use	71 (60–82)	59 (58–60)	**0**.**02**
Tanning device use	5 (0–10)	11 (10–11)	**0**.**02**
Sunburn history	43 (30–56)	47 (46–48)	0.55

The data are presented as percentage (95% confidence interval). Values in bold indicate statistical significance (*P* <0.05). ^a^Includes all respondents with a prior diagnosis of skin cancer, regardless of time since diagnosis. Individuals without a history of skin cancer serve as the reference group.

There was no significant difference in terms of other sun-related behaviours. Overall, 43% of the group with a history of skin cancer reported at least one sunburn. Most of the respondents with a history of skin cancer and those without practised sun avoidance [78% (95% CI 68–88) and 70% (95% CI 69–71), respectively; *P* = 0.10]. Secondary sensitivity analyses with individuals who were not White excluded from the reference group demonstrated similar results, except for a nonsignificant difference in sunscreen use between the two groups.

After stratifying by time elapsed since diagnosis, the majority of adults in all groups used sunscreen, sought shade or avoided sun, and avoided sunburns and tanning devices (Table 3). Rao–Scott χ^2^ analysis revealed significant differences in behaviour between the groups only for protective clothing use, with higher prevalence among individuals 1–4 years (40%; 95% CI 20–60) and ≥10 years (43%; 95% CI 26–61) from diagnosis compared with those without a history of skin cancer (18%; 95% CI 17–19) (*P* < 0.01).

**Table 3:** Prevalence of skin cancer protective behaviours among adults with a history of skin cancer since first diagnosis of skin cancer (weighted), National Health Interview Survey 2005–15

Behaviour	No skin cancer diagnosis(*n* = 61 383 730)	History of skin cancer: time since diagnosis			*P*-value
		1–4 years(*n* = 115 743)	5–9 years(*n* = 43 854)	10–100 years(*n* = 146 871)	
Number	61 383 730	115 743	43 854	146 871	
Sun avoidance^a^	70 (69–71)	85 (71–98)	50 (17–84)	81 (68–95)	0.10
Protective clothing^b^	18 (17–19)	40 (20–60)	22 (0–49)	43 (26–61)	**<0.01**
Sunscreen use	59 (58–60)	75 (58–92)	82 (59–100)	65 (50–80)	0.11
Tanning device use	11 (10–11)	0	8 (0–20)	7 (0–17)	0.28
Sunburn history	47 (46–48)	39 (19–60)	71 (41–100)	37 (19–55)	0.31

The data are presented as percentage (95% confidence interval). Values in bold indicate statistical significance for a *P*-value < 0.05. ^a^Sun avoidance refers to seeking shade when outside on a warm sunny day for >1 h. ^b^Protective clothing indicates use of long pants or clothing that reaches the ankles, long-sleeved shirts or hats when outside on a warm sunny day for >1 h.

Sun avoidance was highest in the group 1–4 years since diagnosis (85%; 95% CI 71–98) and lowest in the 5–9 years group (50%; 95% CI 17–84), while sunscreen use peaked within the first decade after diagnosis, ranging from 75% (95% CI 58–92; 1–4 years) to 82% (95% CI 59–100; 5–9 years). The prevalence of sunburns ranged widely across survivor groups, from 37% (95% CI 19–55; 10–100 years) to 71% (95% CI 41–100; 5–9 years). After participants who were not White were excluded from the reference group, the secondary sensitivity analysis yielded similar results.

In multivariate analysis, after adjusting for demographic covariates, individuals with >10 years since skin cancer diagnosis and 1–4 years since skin cancer diagnosis were significantly more likely to report use of sun-protective clothing than the reference group without skin cancer diagnosis [adjusted OR 2.42 (95% CI 1.22–4.79) for >10 years; adjusted OR 3.17 (95% CI 1.35–7.46) for 1–4 years]. Due to low event counts of tanning device use across the groups ORs could not be calculated. There were no differences in the adjusted prevalence ORs of other behaviours in any of the skin cancer history groups compared with the reference group (Table 4).

**Table 4:** Unadjusted and adjusted multivariate odds ratios (ORs) of skin cancer protective behaviours since first diagnosis of skin cancer (weighted), National Health Interview Survey 2005–15

Behaviour	No skin cancer diagnosis	History of skin cancer: time since diagnosis		
		1–4 years	5–9 years	10–100 years
Sun avoidance^a^				
Unadjusted OR	1 (reference group)	2.40 (0.84–6.86)	0.44 (0.11–1.72)	1.88 (0.78–4.50)
		*P* = 0.10	*P* = 0.24	*P* = 0.16
Adjusted OR	1 (reference group)	2.35 (0.84–6.59)	0.44 (0.10–1.89)	1.70 (0.72–4.03)
		*P* = 0.10	*P* = 0.27	*P* = 0.23
Protective clothing^b^				
Unadjusted OR	1 (reference group)	**3.07 (1.32–7.14)**	1.26 (0.25–6.30)	**3.46 (1.70–7.04)**
		***P*** **<** **0.01**	*P* = 0.77	***P*** **<** **0.01**
Adjusted OR	1 (reference group)	**3.17 (1.35–7.46)**	0.91 (0.19–4.26)	**2.42 (1.22–4.79)**
		***P*** **<** **0.01**	*P* = 0.90	***P*** **=** **0.01**
Sunscreen use				
Unadjusted OR	1 (reference group)	2.09 (0.84–5.22)	3.12 (0.71–13.7)	1.32 (0.68–2.59)
		*P* = 0.11	*P* = 0.13	*P* = 0.41
Adjusted OR	1 (reference group)	1.78 (0.80–3.96)	3.05 (0.84–11.1)	1.29 (0.63–2.64)
		*P* = 0.16	*P* = 0.09	*P* = 0.49
Tanning device use^c^	NA	NA	NA	NA
Sunburn history				
Unadjusted OR	1 (reference group)	0.73 (0.31–1.73)	2.80 (0.65–12.2)	0.68 (0.31–1.46)
		*P* = 0.48	*P* = 0.17	*P* = 0.32
Adjusted OR	1 (reference group)	0.74 (0.27–2.04)	3.15 (0.70–14.1)	0.93 (0.43–1.98)
		*P* = 0.56	*P* = 0.13	*P* = 0.85

Bold values indicate statistical significance. Adjusted ORs are adjusted for age, sex, region, health insurance, alcohol use, smoking status, education, income, and personal and family history of skin cancer. NA, not applicable. ^a^Sun avoidance refers to seeking shade when outside on a warm sunny day for >1 h. ^b^Protective clothing indicates use of long pants or clothing that reaches the ankles, long-sleeved shirts or hats when outside on a warm sunny day for >1 h. ^c^There were not enough events in the tanning device category to perform an analysis.

After excluding individuals who were not White from the reference group, there were no additional differences in behaviours between groups, but the use of sun-protective clothing for adults with >10 years since diagnosis was no longer significant (Table S2; see Supporting Information).

## Discussion

In this study, we found that adults with a history of skin cancer reported significantly higher use of sun-protective behaviours, such as protective clothing and sunscreen use, compared with the cohort without a history of skin cancer (Table 2). Although adults with a history of skin cancer were more likely to be White than the reference group in our sample, secondary analysis showed consistent results. Despite these increased protective behaviours, over one-third of adults with a history of skin cancer reported at least one sunburn over the last year, slightly higher than previous estimates of 20% of survivors of melanoma with recent sunburns.^5^ These findings suggest that a skin cancer diagnosis could act as a critical motivator for certain sun-protection practices, with additional continued challenges in avoiding sun exposure and proper use of protective measures.

A key strength of this study is the examination of photo­protective behaviours over time since skin cancer diagnosis, which revealed behavioural fluctuations across time strata. Sun avoidance was highest in the group 1–4 years since diagnosis (85%) and dropped to 50% in the 5–9 years group, before partially recovering afterwards. Sunscreen use showed a similar pattern, peaking in the first decade after diagnosis (75–82% vs. 59% in controls) and declining modestly at ≥10 years. Sunburn prevalence also varied, with the highest prevalence reported at 5–9 years (71%). These differences did not reach statistical significance under the Rao–Scott χ^2^ test, likely due to wider CIs. In contrast, use of protective clothing showed a significant association with time since diagnosis (*P* < 0.01), with higher rates at both 1–4 years (40%) and ≥10 years (43%) compared with control individuals (18%).

These findings may reflect early postdiagnosis vigilance that diminishes with time before stabilizing, highlighting the potential need for reinforced sun-safety counselling at routine follow-up visits, years after the initial diagnosis. In our adjusted multivariate analysis, these findings persisted: survivors of skin cancer 1–4 years from diagnosis and those with ≥10 years since diagnosis had significantly higher odds of wearing protective clothing compared with those without a history of skin cancer. Most of the other assessed behaviours, such as sunscreen use and sun avoidance, did not demonstrate significant differences in ORs, although they demonstrated directionally consistent trends towards greater photoprotection early after diagnosis. Indoor tanning use was rare among survivors and lowest in the early postdiagnosis period, supporting effective avoidance of this high-risk behaviour.

A prior study found that men with a history of skin cancer had a higher prevalence of sunburns than population controls, suggesting that some individuals with a history of skin cancer may not perceive a significant risk for additional skin cancers, and identification of these patients is critical.^11^ The Sun Protection Behavior Checklist from Memorial Sloan Kettering, which includes items similar to those in the NHIS, may aid dermatologists in identifying those with a history of skin cancer who could benefit from more intensive photoprotection counselling.^12^

Younger patients with prior skin cancer, despite concerns about future skin cancers, often engage in less protective behaviours, reinforcing the need for age-specific intervention and assessment.^13,14^ Notably, among survivors of childhood cancer, those who perceived themselves as more susceptible to skin cancer, or who reported a lighter skin type or proclivity to sunburns, were more likely to adopt photoprotective behaviours. A study of Swedish adults found that lighter skin and a personal history of skin cancer were associated with sun-protective behaviours, while family history and childhood sunburns were not.^15,16^ These findings suggest that reinforcing personal risk could be particularly effective in motivating behavioural changes in at-risk populations, potentially with the use of UV index as a practical tool for decision making around sun exposure.^17^

The prevalence of use of protective clothing was consistently lower than for other photoprotective behaviours, perhaps due to issues such as cost, inconvenience and social acceptability.^15^ Despite these barriers, UV protection factor clothing should be considered as a recommendation for patients with a history of skin cancer, as this type of clothing can potentially offer superior and reliable protection against UV radiation compared with some sunscreen formulations.^18,19^ Although sunscreen use was high, many respondents still experienced sunburn, possibly due to improper application, not reapplying or overestimation of protection by users.^20,21^ Importantly, reliance on sunscreen alone may foster a sense of security, potentially leading to prolonged sun exposure.^22,23^

This study has several limitations. The NHIS data assessed include historical data from 2005 to 2015; however, this represents the most recent comprehensive collection of sun-protective behaviours on a national scale in the USA. The NHIS also asks about photoprotective behaviours specifically in the context of being outdoors for >1 h on a sunny day, which may not reflect respondents’ day-to-day sun-protection habits. Additionally, the cross-sectional nature of the study limits causal inferences, and self-reported data introduce the potential for recall bias.

Our findings underscore the need for sustained long-term behavioural interventions among individuals with a history of skin cancer. Dermatologists play a crucial role in reinforcing photo­protection, and counselling strategies should account for the potential attenuation of protective habits over time.

## Supplementary Material

vzag024_Supplementary_Data

## References

[1] Roky AH, Islam MM, Ahasan AMF et al Overview of skin cancer types and prevalence rates across continents. Cancer Pathog Ther 2024; 3:89–100. 40182119 10.1016/j.cpt.2024.08.002 PMC11963195

[2] Bradford PT, Freedman DM, Goldstein AM et al Increased risk of second primary cancers after a diagnosis of melanoma. Arch Dermatol 2010; 146:265–72. 20231496 10.1001/archdermatol.2010.2 PMC3076705

[3] Chattopadhyay S, Hemminki A, Försti A et al Second primary cancers in patients with invasive and *in situ* squamous cell skin carcinoma, Kaposi sarcoma, and Merkel cell carcinoma: role for immune mechanisms? J Invest Dermatol 2020; 140:48–55. 31288011 10.1016/j.jid.2019.04.031

[4] Fischer AH, Wang TS, Yenokyan G et al Sunburn and sun-protective behaviors among adults with and without previous nonmelanoma skin cancer (NMSC): a population-based study. J Am Acad Dermatol 2016; 75:371–9. 27198078 10.1016/j.jaad.2016.02.1236 PMC4958558

[5] Vogel RI, Strayer LG, Engelman L et al Sun exposure and protection behaviors among long-term melanoma survivors and population controls. Cancer Epidemiol Biomarkers Prev 2017; 26:607–13. 28254810 10.1158/1055-9965.EPI-16-0854 PMC5380539

[6] Flohil SC, van der Leest RJ, Arends LR et al Risk of subsequent cutaneous malignancy in patients with prior keratinocyte carcinoma: a systematic review and meta-analysis. Eur J Cancer 2013; 49:2365–75. 23608733 10.1016/j.ejca.2013.03.010

[7] Heckman CJ, Manne SL, Kashy DA et al Correlates of sun protection behaviors among melanoma survivors. BMC Public Health 2021; 21:882. 33962615 10.1186/s12889-021-10951-1 PMC8105954

[8] Tollosa DN, Holliday E, Hure A et al Multiple health behaviors before and after a cancer diagnosis among women: a repeated cross-sectional analysis over 15 years. Cancer Med 2020; 9:3224–33. 32134568 10.1002/cam4.2924 PMC7196049

[9] Sharp K, Olafsdottir EJ, Sahni DR et al Survival of patients with basal cell carcinoma, squamous cell carcinoma, and squamous cell carcinoma *in situ*: a whole population study. J Am Acad Dermatol 2024; 90:91–7. 37758026 10.1016/j.jaad.2023.09.044

[10] Parsons VL, Moriarity C, Jonas K et al Design and estimation for the national health interview survey, 2006-2015. Vital Health Stat 2 2014; 165:1–53. 24775908

[11] Ottwell R, Cook C, Greiner B et al Lifestyle behaviors and sun exposure among individuals diagnosed with skin cancer: a cross-sectional analysis of 2018 BRFSS data. J Cancer Surviv 2021; 15:792–8. 33230725 10.1007/s11764-020-00971-y

[12] Cowen EA, Veldhuizen IJ, Klassen AF et al Sun protection behaviour checklist for targeted counselling in skin cancer patients. Australas J Dermatol 2022; 63:392–4. 35460573 10.1111/ajd.13845 PMC9946314

[13] Domínguez Bueso DL, González Ruiz LM, Mondragón Márquez LI et al Skin cancer survivorship and sun protection behaviors in the United States. Photodermatol Photoimmunol Photomed 2024; 40:e12933. 38288776 10.1111/phpp.12933

[14] Hawkins DM, Jacobsen G, Johnson CC et al Self-reported quality of life after skin cancer in young adults. J Dermatolog Treat 2015; 26:357–60. 25490454 10.3109/09546634.2014.991671

[15] Fluehr M, Kwok G, Stapleton JL et al Factors associated with sun protection behaviors among childhood cancer survivors. J Pediatr Hematol Oncol 2023; 45:e323–7. 36706312 10.1097/MPH.0000000000002618 PMC10038824

[16] Karlsson O, Hagberg O, Nielsen K et al Difference in sun exposure habits between individuals with high and low risk of skin cancer. Dermatol Pract Concept 2021; 11:e2021090. 34631260 10.5826/dpc.1104a90 PMC8480439

[17] Snyder AN, Litchman GH, Plante JG et al Ultraviolet index counseling as a primary prevention strategy by US dermatologists. JAAD Int 2020; 1:48–9. 34409321 10.1016/j.jdin.2020.04.006 PMC8361887

[18] Berry EG, Bezecny J, Acton M et al Slip versus slop: a head-to-head comparison of UV-protective clothing to sunscreen. Cancers (Basel) 2022; 14:542. 35158810 10.3390/cancers14030542 PMC8833350

[19] Patel SP, Chien AL. Sun protective clothing and sun avoidance: the most critical components of photoprotection in patients with melanoma. Dermatol Surg 2021; 47:333–7. 32991331 10.1097/DSS.0000000000002794

[20] Kim S, Carson KA, Chien AL. Prevalence and correlates of sun protections with sunburn and vitamin D deficiency in sun-sensitive individuals. J Eur Acad Dermatol Venereol 2020; 34:2664–72. 32453868 10.1111/jdv.16681 PMC7688502

[21] Dang J, Reserva J, Tung-Hahn E et al Sunscreen application technique amongst patients with a history of skin cancer. Arch Dermatol Res 2020; 312:739–46. 32929600 10.1007/s00403-020-02131-9

[22] Autier P . Sunscreen abuse for intentional sun exposure. Br J Dermatol 2009; 161:40–5. 10.1111/j.1365-2133.2009.09448.x 19775356

[23] Petersen B, Wulf HC. Application of sunscreen—theory and reality. Photodermatol Photoimmunol Photomed 2014; 30:96–101. 24313722 10.1111/phpp.12099

