# Supplementary material for: Sun-protective behaviours in US adults with a history of skin cancer: a cross-sectional National Health Interview Survey study
Source: Skin Health Dis. 2026 May 15;6(4):431–7. doi: 10.1093/skinhd/vzag024 (PMC13425089; doi:10.1093/skinhd/vzag024)
Supplement: vzag024_Supplementary_Data [file vzag024_supplementary_data.zip › NHIS_Supplementary Table 2.docx]

**Supplementary Table 2.** Unadjusted and adjusted multivariate odds ratios of skin cancer protective behaviors since first diagnosis of skin cancer (weighted) with non-white individuals excluded from reference group, National Health Interview Survey 2005 to 2015

|  | Individuals with no history of skin cancer diagnosis | Individuals with 1-4 years since skin cancer diagnosis | Individuals with 5-9 years since skin cancer diagnosis | Individuals with 10-100 years since skin cancer diagnosis |
| --- | --- | --- | --- | --- |
| Sun avoidance^1^  Unadjusted OR  aOR* | 1 [Ref]  1 [Ref] | 2.75 (0.89, 8.46) (p=0.08)  2.58 (0.85-7.82) (p=0.09) | 0.46 (0.12, 1.78) (p=0.26)  0.43 (0.10-1.88) (p=0.26) | 1.81  (0.74, 4.44) (p=0.19)  1.58 (0.65-3.84) (p=0.31) |
| Protective clothing^2^  Unadjusted OR  aOR* | 1 [Ref]  1 [Ref] | **3.32 (1.42, 7.76) (p<0.01)**  **3.20 (1.35-7.61) (p<0.01)** | 1.33 (0.27, 6.62) (p=0.73)  0.87 (0.18-4.30) (p=0.87) | **3.18 (1.48, 6.85) (p<0.01)**  2.08 (0.98-4.40) (p=0.06) |
| Sunscreen use  Unadjusted OR  aOR* | 1 [Ref]  1 [Ref] | 1.80 (0.69, 4.66) (p=0.23)  1.85 (0.80-4.28) (p=0.15) | 2.53 (0.58, 11.06) (p=0.22)  3.15 (0.86-11.49) (p=0.08) | 1.17 (0.58, 2.36) (p=0.67)  1.37 (0.64-2.93) (p=0.41) |
| Tanning device use^3^  Unadjusted OR  aOR* | N/A | N/A | N/A | N/A |
| Sunburn history  Unadjusted OR  aOR* | 1 [Ref]  1 [Ref] | 0.61 (0.26, 1.44) (p=0.26)  0.74 (0.26, 2.13) (p=0.58) | 2.26 (0.52, 9.81) (p=0.28)  3.41 (0.77, 15.06) (p=0.83) | 0.55 (0.25, 1.21) (p=0.14)  0.92 (0.42, 2.00) (p=0.10) |

Bold values indicate statistical significance.

*aOR*; Adjusted odds ratio; *CI*, confidence interval; *OR*, odds ratio.

*aOR is adjusted for age, race, sex, region, health insurance, alcohol use, smoking status, education, income, personal and family history of skin cancer

^1^Sun avoidance refers to seeking shade when outside on a warm sunny day for more than one hour.

^2^Protective clothing indicates use of long pants or clothing that reaches the ankles, long-sleeved shirts, or hats when outside on a warm sunny day for more than one hour.

^3^There were very few events in the tanning device category, so we opted to forego logistic regression for this prompt.
