# Supplementary material for: Sun-protective behaviours in US adults with a history of skin cancer: a cross-sectional National Health Interview Survey study
Source: Skin Health Dis. 2026 May 15;6(4):431–7. doi: 10.1093/skinhd/vzag024 (PMC13425089; doi:10.1093/skinhd/vzag024)
Supplement: vzag024_Supplementary_Data [file vzag024_supplementary_data.zip › NHIS_SupplementaryTable1.docx]

**Supplementary Table 1.** Distribution of responses across original sun-protective behavior categories by skin cancer history and time since diagnosis, National Health Interview Survey 2005 to 2015 (weighted)

|  |  | Always | Most of the time | Sometimes | Rarely | Never |
| --- | --- | --- | --- | --- | --- | --- |
| Use of sun-protective clothing | No history of skin cancer | 706,128 (1.2%) | 1,837,891 (3.0%) | 8,525,320 (13.9%) | 18,807,755 (30.6%) | 30,235,512 (49.3%) |
|  | 1-4 years since skin cancer diagnosis | 0 (0.0%) | 14,606 (12.6%) | 32,803 (28.3%) | 39,079 (33.8%) | 29,975 (25.9%) |
|  | 5-9 years since skin cancer diagnosis | 0 (0.0%) | 4,072 (9.3%) | 5,472 (12.5%) | 14,515 (33.1%) | 19,795 (45.1%) |
|  | 10+ years since skin cancer diagnosis | 9,407 (6.4%) | 5,171 (3.5%) | 48,929 (33.3%) | 49,831 (33.9%) | 33,533 (aps22.8%) |
| Sunscreen use | No history of skin cancer | 12,172,099 (19.8%) | 10,169,272 (16.6%) | 13,621,067 (22.2%) | 6,767,819 (11.0%) | 17,475,360 (28.5%) |
|  | 1-4 years since skin cancer diagnosis | 49,144 (42.4%) | 34,912 (30.2%) | 2,443 (2.1%) | 8,201 (7.1%) | 21,043 (18.2%) |
|  | 5-9 years since skin cancer diagnosis | 21,250 (48.4%) | 6,452 (14.7%) | 8,052 (18.4%) | 0 (0.0%) | 8,100 (18.5%) |
|  | 10+ years since skin cancer diagnosis | 43,869 (29.9%) | 25,904 (17.6%) | 25,928 (17.7%) | 23,407 (15.9%) | 27,763 (18.9%) |
| Sun avoidance/shade seeking | No history of skin cancer | 4,898,927 (8.0%) | 14,150,910 (23.1%) | 23,764,205 (38.7%) | 11,083,063 (18.1%) | 6,168,430 (10.0%) |
|  | 1-4 years since skin cancer diagnosis | 12,137 (10.5%) | 39,791 (34.4%) | 46,095 (39.8%) | 11,711 (10.1%) | 6,009 (5.2%) |
|  | 5-9 years since skin cancer diagnosis | 4,455 (10.2%) | 11,754 (26.8%) | 5,882 (13.4%) | 21,763 (49.6%) | 0 (0.0%) |
|  | 10+ years since skin cancer diagnosis | 22,182 (15.1%) | 51,464 (35.0%) | 45,684 (31.1%) | 24,056 (16.4%) | 3,485 (2.4%) |

^1^ Values represent weighted distributions across original NHIS response categories prior to dichotomization.
